# Integrated microRNA and mRNA Profiling in the Corneal Endothelium of Fuchs Endothelial Corneal Dystrophy

**DOI:** 10.3390/ijms272110044

**Published:** 2026-11-09

**Authors:** Dmitriy Ryazantsev, Elena Sharova, Denis Gudkov, Anastasia Voronkova, Boris Malyugin, Olga Antonova, Alexandra Belodedova, Liubov Skorodumova

**Affiliations:** 1 Medical Genomics Laboratory, Lopukhin Federal Research and Clinical Center of Physical, Chemical Medicine of the Federal Medical Biological Agency, Moscow 119435, Russia; rezan1998mail@gmail.com (D.R.); sharova78@gmail.com (E.S.); anast.voronkova@gmail.com (A.V.); 2 Department of Pharmacology, Federal State Budgetary Educational Institution of Higher Education “Russian University of Medicine”, The Ministry of Health of the Russian Federation, Moscow 127006, Russia; gudkovdenis@gmail.com; 3 The Jules Stein Eye Institute, University of California at Los Angeles, 200 Stein Plaza, Los Angeles, CA 90095, USA; bmalyugin@mednet.ucla.edu; 4 Department of Anterior Segment Transplant and Optical Reconstructive Surgery, S. Fyodorov Eye Microsurgery Complex Federal State Institution, Moscow 127486, Russia; antonova.mntk@gmail.com (O.A.); ale.belodedova@gmail.com (A.B.)

**Keywords:** Fuchs endothelial corneal dystrophy, microRNA, corneal endothelium, miR-29

## Abstract

MicroRNAs (miRNAs) are endogenous non-coding RNAs that post-transcriptionally modulate gene expression, playing roles in both development and disease pathogenesis. This study specifically investigates the differential miRNA expression in human FECD endothelium compared to healthy controls. By integrating this miRNAome profile with our existing transcriptomic background, we aimed to identify candidate miRNA–target gene regulatory relationships potentially relevant to FECD. NanoString nCounter miRNA profiling was performed on corneal endothelium from FECD patients and controls. Differential expression analysis was conducted, followed by correlation of miRNAs with transcriptomic data from matched samples. Functional enrichment was performed for negatively correlated miRNA–mRNA pairs to identify pathways relevant to FECD pathogenesis. We found 98 differentially expressed miRNAs between FECD and controls. Correlation analysis revealed negatively correlated candidate miRNA–target gene pairs, highlighting potential regulatory interactions. Enrichment analysis of target genes implicated PI3K-AKT, TNF, AGE-RAGE, and focal adhesion pathways as part of FECD molecular alterations. Several dysregulated miRNAs (e.g., miR-302 and miR-630) were associated with processes such as extracellular matrix remodeling, apoptosis, and inflammatory signaling, consistent with key pathogenic features of FECD. This exploratory study provides new insights into the pathogenesis of FECD by integrating miRNA profiling with a transcriptomic background. This combined analysis highlights candidate miRNA–target associations identified within the FECD group and places them into potentially regulatory pathways relevant to endothelial degeneration. The observed miR-29 expression trends are hypothesis-generating; however, the miR-29 family did not meet the prespecified FDR threshold and was not retained in the final correlation network, precluding confirmation of its contribution to ECM remodeling in our cohort. In parallel, comparison with earlier array-based studies revealed inconsistencies that may partly reflect differences in platforms and cohorts; these discrepancies warrant further investigation in independent cohorts. Finally, the analysis of mirtronic host genes uncovered unstable correlations, suggesting that array-based measurements alone are insufficient for reliable miRNA quantification. Together, these findings refine our understanding of FECD-related networks, resolve some earlier contradictions, and open directions for more robust validation and therapeutic exploration.

## 1. Introduction

Fuchs endothelial corneal dystrophy (FECD) is the most common form of corneal endothelial dystrophy. It is characterized by progressive loss of corneal endothelial cells, thickening of Descemet’s membrane, and the development of excrescences known as guttae. A critical function of the corneal endothelium is to actively pump fluid out of the corneal stroma, thereby maintaining corneal deturgescence and transparency [1]. Progressive endothelial cell loss compromises this barrier and pump function, leading to stromal edema, reduced corneal clarity, and, in advanced disease, painful epithelial bullae and visual impairment.

FECD presents in two forms: early-onset and late-onset. The early-onset form is rare, with clinical manifestations generally emerging in the second decade of life [2,3]. In contrast, the late-onset form is commonly diagnosed in individuals in their sixth decade or later [4,5,6,7]. This study focuses on the late-onset form. A marked female predominance is observed in late-onset FECD, with an approximate male-to-female ratio of 1:3 [4,7,8,9]. Genetic etiology of the late-onset FECD is complex. The most common established genetic cause is the expansion of the CTG18.1 trinucleotide repeat in the *TCF4* gene [10,11]. However, the frequency of this expansion varies substantially among populations, ranging from 17% to 81.1% of patients depending on ethnicity [12]. Variants in several other genes have also been proposed as contributors to late-onset FECD, although their pathogenic roles remain unconfirmed or disputed [13,14,15]. Thus, the search for genetic causes of FECD not associated with CTG18.1 repeat expansion is in progress.

The pathogenesis of FECD remains incompletely understood. Immunohistochemical, transcriptomic, and proteomic analyses have demonstrated pronounced alterations in the composition of the Descemet’s membrane and corneal endothelial cells in FECD [16,17,18,19,20,21,22]. In particular, corneal endothelial cells exhibit upregulated expression of extracellular matrix (ECM)-associated genes [18,21,23,24,25,26]. In advanced disease, corneal endothelial cells progressively lose their characteristic hexagonal morphology and acquire a fibroblast-like phenotype, suggesting a shift toward epithelial–mesenchymal transition (EMT) [14]. Oxidative stress and unfolded protein response are recognized as major pathogenic factors contributing to endothelial cell dysfunction and loss in FECD [14,27,28]. Since the CTG18.1 trinucleotide repeat expansion is the primary established etiology in FECD, most studies have focused on its pathogenic effects. Two main hypotheses have been proposed to explain CTG18.1 toxicity: (1) RNA-mediated toxicity from the expansion, disrupting alternative splicing [24,29,30,31,32,33,34,35]; and (2) impaired transcription of *TCF4* variants, whose promoters lie adjacent to the repeats, shifting TCF4 protein isoform abundance [36,37,38]. Nevertheless, transcriptomic profiles from FECD patients with and without CTG18.1 expansion show substantial overlap in differentially expressed genes [24,38,39]. This suggests shared molecular pathways or regulatory networks drive most late-onset FECD cases, irrespective of expansion status.

Research on small RNA regulation in corneal endothelium, particularly in FECD, remains limited. MicroRNAs (miRNAs) are small, non-coding RNA molecules that regulate gene expression post-transcriptionally. By binding to complementary sequences within target mRNAs, miRNAs promote translational repression or mRNA degradation [40]. It is estimated that over 60% of human protein-coding genes are regulated by miRNAs [41]. Early evidence for the involvement of miRNAs in corneal endothelial biology came from the identification of a miR-184 mutation in EDICT syndrome, which is characterized by endothelial dystrophy, iris hypoplasia, congenital cataract, and stromal thinning [42]. Subsequent detection of miRNAs in aqueous humor, a fluid that directly bathes the corneal endothelium, further supported their potential relevance to corneal endothelial homeostasis [43]. In addition, miRNA dysregulation has been observed in the corneal endothelium of aging mice, including miRNAs associated with TGF-β signaling and oxidative phosphorylation—pathways also implicated in FECD [44].

Pivotal studies have highlighted the potential pathogenic role of miRNAs in FECD. In 2014, Matthaei et al. demonstrated widespread miRNA downregulation accompanied by reduced DICER1 mRNA expression in the corneal endothelium of FECD patients compared with healthy controls, with a pronounced reduction in hsa-miR-29b [45]. The miR-29 family (miR-29a/b/c) regulates ECM homeostasis and fibrogenesis. hsa-miR-29b overexpression led to a decrease in COL1A1, COL4A1, and LAMC1 mRNA levels and significantly reduced ECM protein production [46]. These findings are consistent with in vitro and murine evidence that miR-29 promotes wound healing through inhibition of TGF-β signaling and repression of laminin expression [47,48,49]. Moreover, miR-29a and miR-29b exert anti-angiogenic effects by targeting VEGFA and PDGFB, and their deficiency exacerbates pathological neovascularization in ocular models. Together, these data suggest that miRNA dysregulation may contribute not only to ECM remodeling and fibrosis, but also to other pathological processes relevant to advanced FECD.

Although previous studies have provided important evidence for miRNA dysregulation in FECD, several questions remain unresolved. In particular, how differentially expressed miRNAs may regulate disease-relevant mRNA targets and pathways, and whether the previously reported global reduction in miRNA abundance is reproducible in independent patient cohorts.

Therefore, the aim of this study was to characterize miRNA-mediated regulatory alterations in the corneal endothelium of patients with late-onset FECD. Specifically, we aimed to (1) identify miRNAs with differential expression between FECD corneal endothelium and healthy control tissue; (2) integrate miRNA expression profiles with matched mRNA transcriptomic data to identify potential miRNA–mRNA regulatory relationships and disease-relevant pathways; and (3) determine whether the previously reported global downregulation of miRNAs in FECD corneal endothelium is reproduced in our cohort.

## 2. Results

### 2.1. Experimental Design

For microRNA analysis, total RNA and small RNA fractions were isolated from the corneal endothelium of patients with FECD and control donors. The initial miRNA profiling cohort comprised 16 FECD and 11 control samples. Five samples were subsequently excluded due to potentially unreliable transcriptomic data (three control samples, namely C_209, C_210, and C_211, with suspected epithelial–mesenchymal transition, and two FECD samples, namely Dfu_209 and Dfu_223, with a significant admixture of vascular endothelial cells), resulting in a final dataset of 14 FECD and eight control samples. The final dataset comprised two males and 12 females in the FECD group, with 11 of 14 participants carrying expanded CTG18.1 repeats (>40), and six males and two females in the control group without expanded samples (Table 1). Sample-level metadata, RIN values, and exclusion reasons for all specimens are provided in Supplementary File S2, and a participant flow diagram is shown in Supplementary File S1.

**Figure 1:**
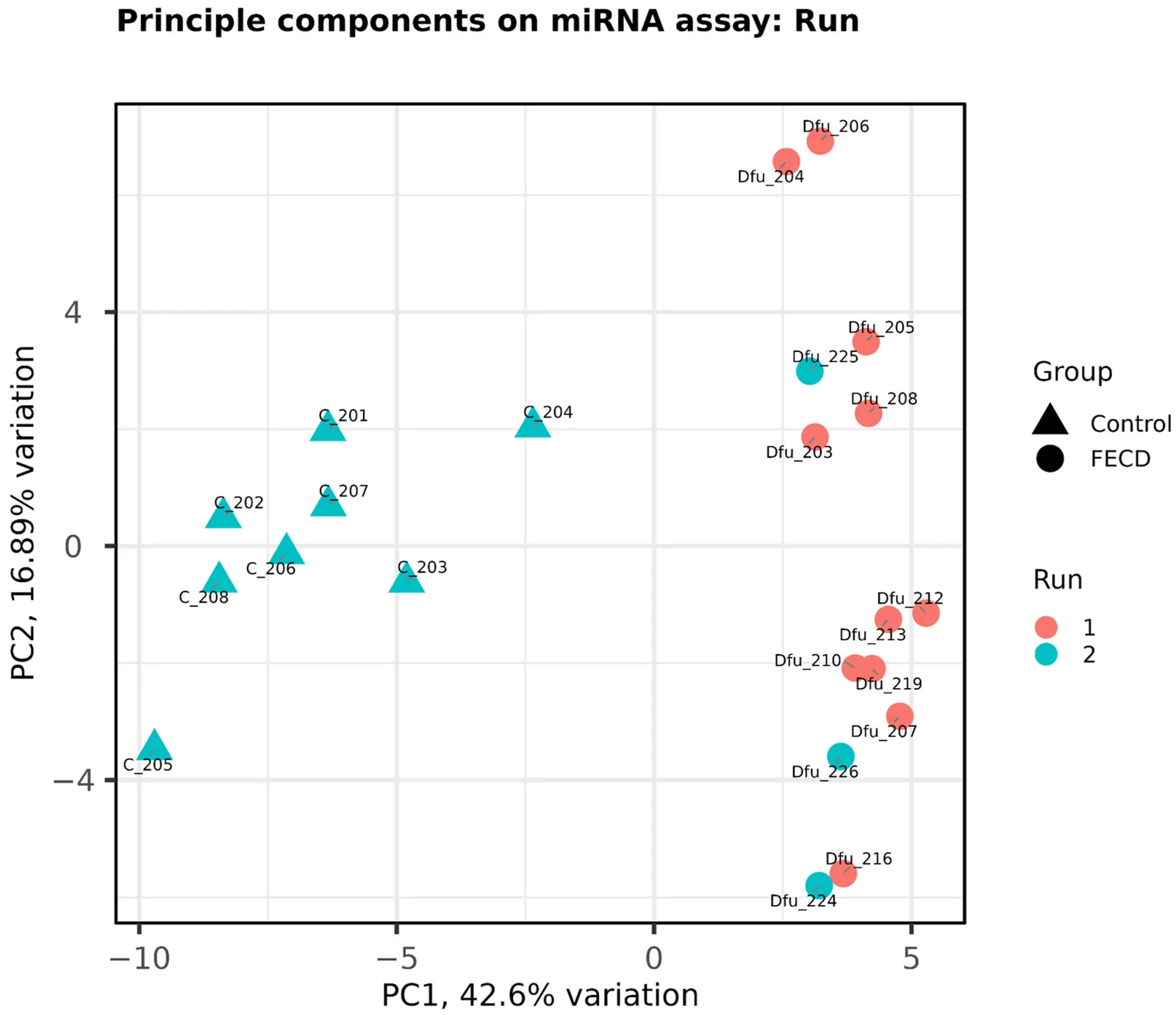
Principal component analysis of the miRNA assay counts. Shape represents disease status: FECD (*n* = 14) and control (*n* = 8). Color represents a different assay microchip (1st and 2nd).

Of these, eight FECD and eight control samples had matched RNA-seq transcriptomic data available and were used for the subsequent miRNA–mRNA correlation analyses. The 653 (82%) non-zero miRNAs proceeded to downstream analysis. To test whether performing miRNA profiling in different instrument runs introduced a strong batch effect, we conducted principal component analysis of the miRNA normalized counts (Figure 1). We showed that samples cluster primarily by disease status rather than by assay chip. Different assay chips did not introduce a strong batch effect. Investigated groups are clearly separated by disease (Figure 1).

### 2.2. miRNA Differential Expression

**Figure 2:**
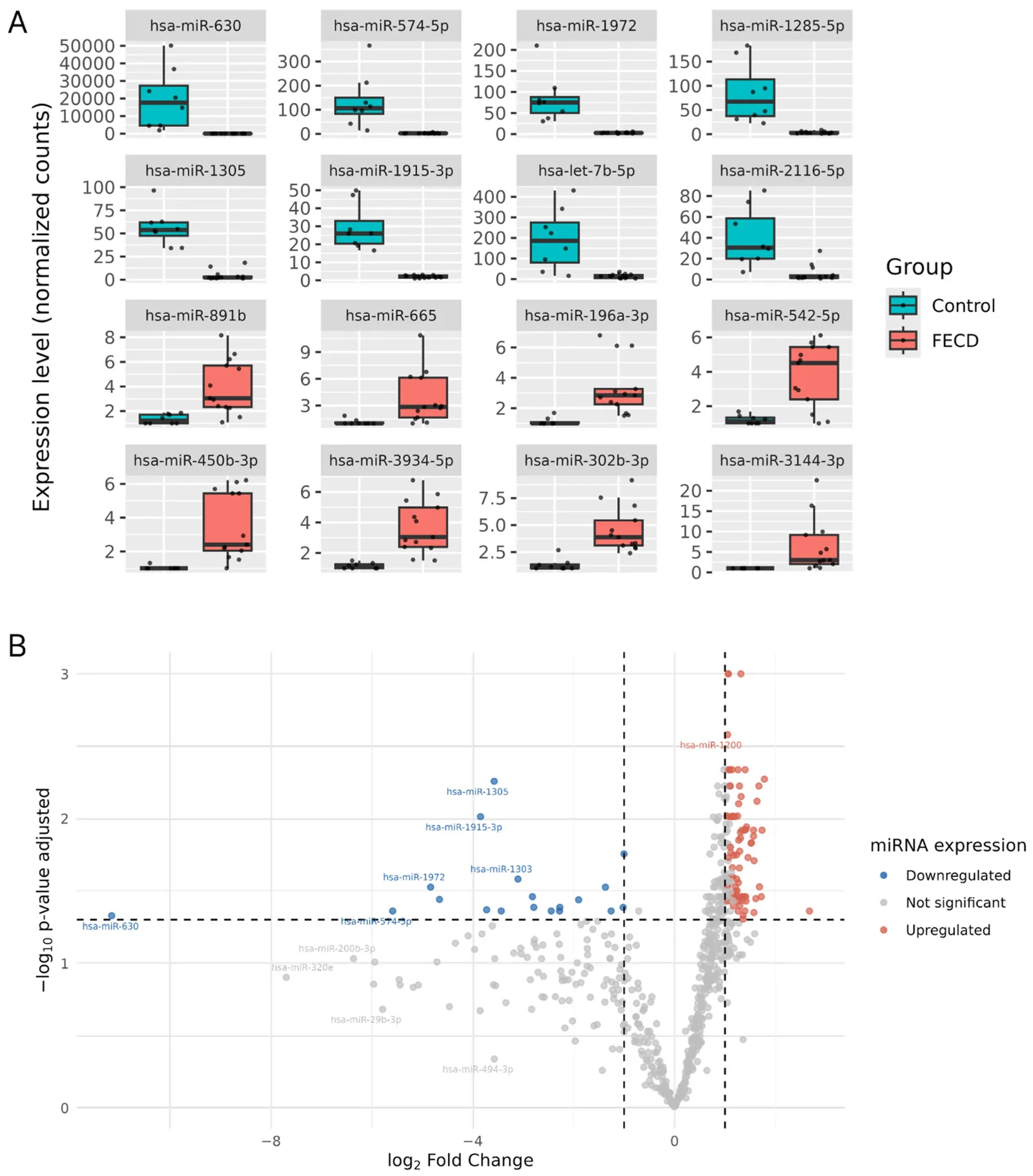
Differentially expressed miRNAs in FECD corneal endothelium. (**A**) Top eight upregulated and downregulated miRNAs, ordered by Fold Change (FDR-adjusted *p* < 0.05). (**B**) Volcano plot illustrating miRNA expression changes; dashed horizontal line indicates FDR-adjusted significance threshold (*p* = 0.05).

Analysis of dataset revealed 98 differentially expressed (DE) microRNAs (FDR < 0.05, |log2FC| > 1), with 79 upregulated and 19 downregulated among them (Figure 2; Supplementary File S3).

Top downregulated DE miRNAs in FECD by Fold Change are hsa-miR-630, hsa-miR-574-5p, hsa-miR-1972, hsa-miR-1285-5p (mean normalized counts in FECD group are 8.62, 2.79, 2.94, and 3.33, respectively). Top upregulated DE miRNAs in FECD by Fold Change are hsa-miR-3144-3p, hsa-miR-302b-3p, hsa-miR-3934-5p, hsa-miR-450b-3p (mean normalized counts in FECD group are 6.37, 4.56, 3.68, and 3.45, respectively).

### 2.3. Correlated miRNA–Target Genes Pairs

To explore possible regulatory miRNAs’ role in FECD, we analyzed negatively correlated miRNA–target genes pairs. After all filtering steps, the control samples retained 464 highly negatively correlated miRNA–target genes pairs, comprising 211 unique target genes and 231 unique miRNAs. In the FECD samples, 249 pairs remained, with 123 unique target genes and 127 unique miRNAs (Supplementary File S4). The most correlated pairs in the FECD group are shown below (Table 2).

**Figure 3:**
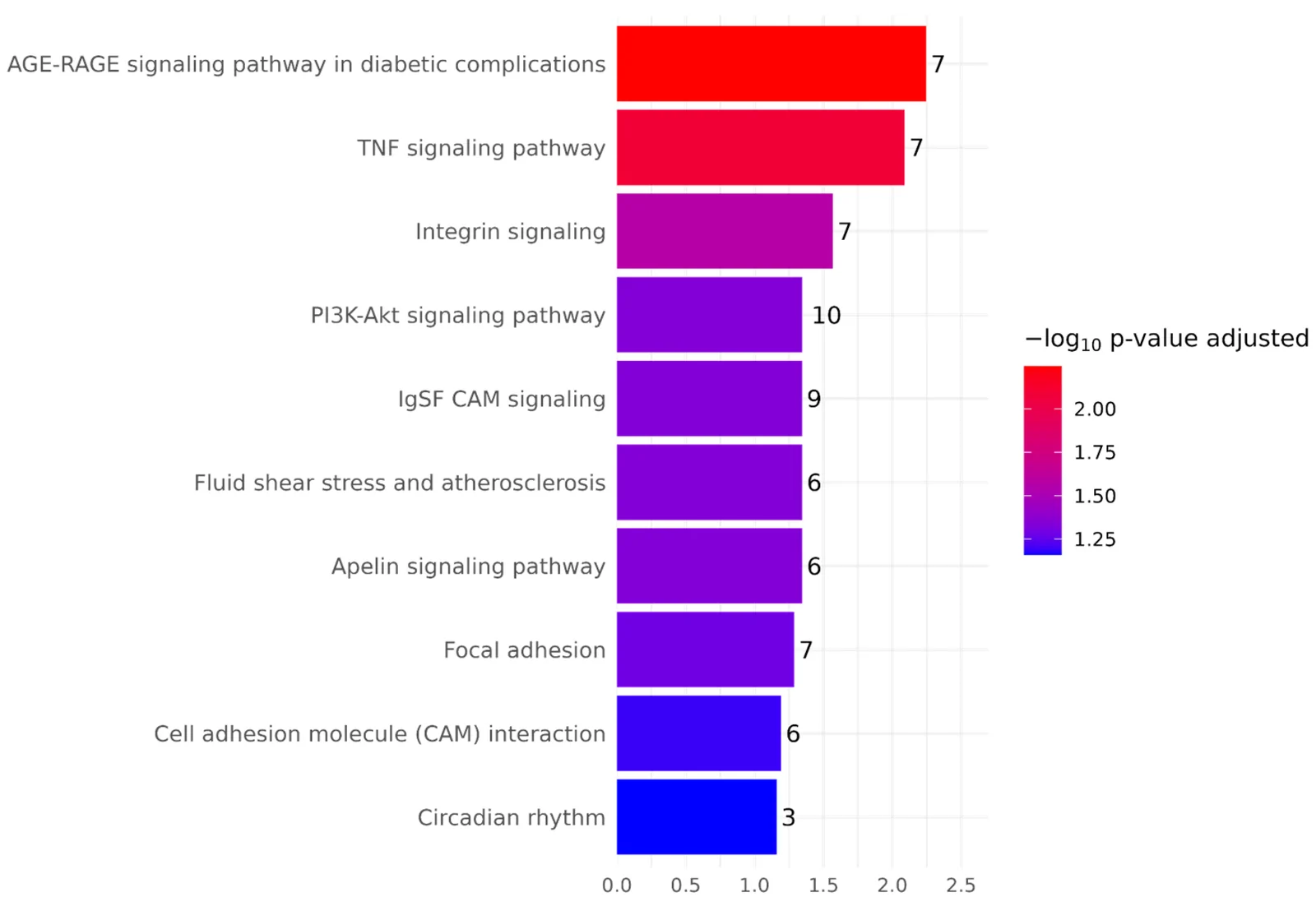
KEGG enrichment pathways with negatively correlated miRNA–target genes in FECD. The digit on the column indicates intersection between pathway genes and possible target genes. Color indicates significance in −log10 *p*-value FDR-adjusted.

**Figure 4:**
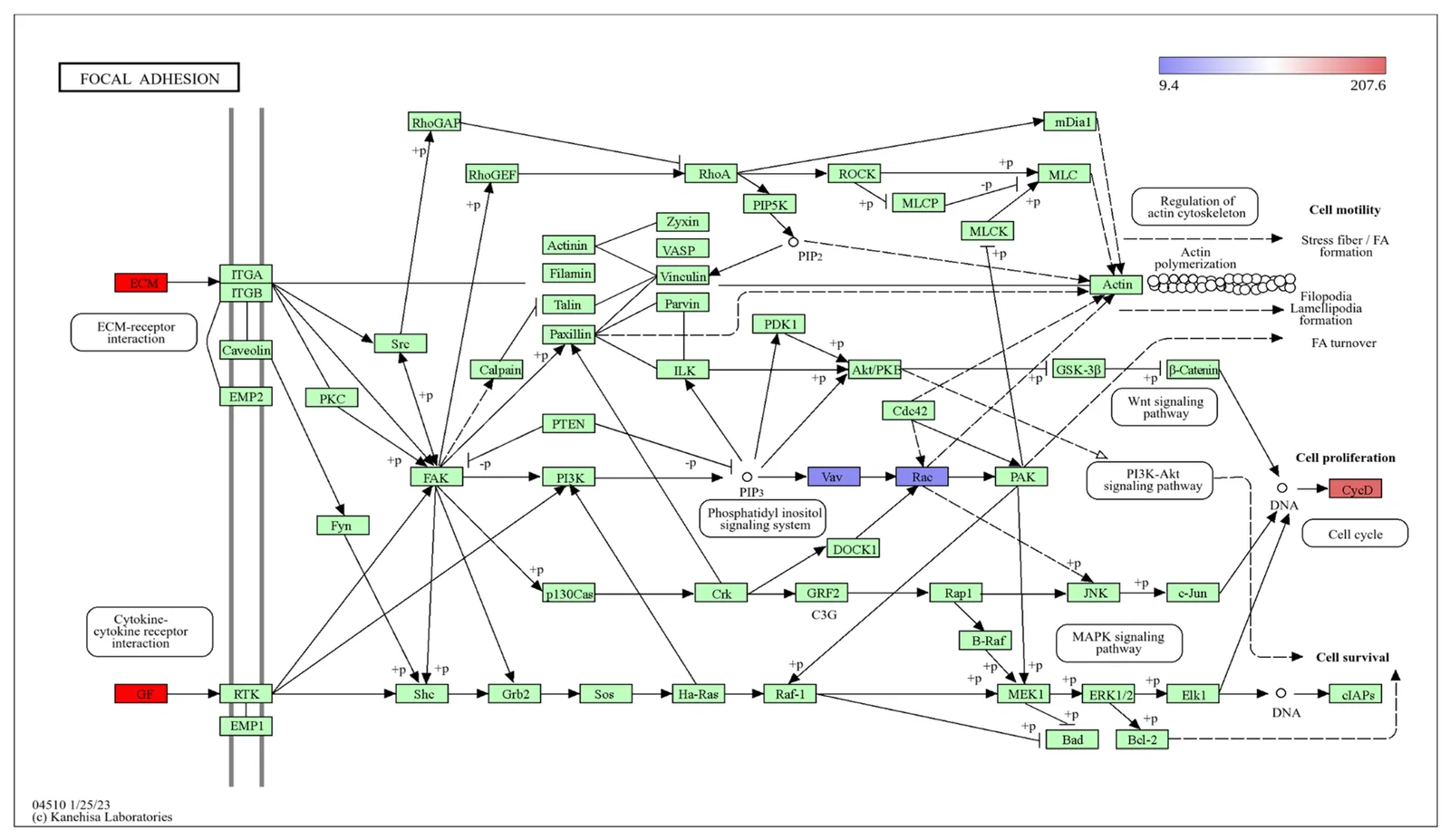
KEGG enrichment card on FECD miRNAs’ target genes showing probable focal adhesion pathway regulation. Genes on cards are colored by CPM-normalized RNA-seq counts.

KEGG pathway analysis of miRNA’s target genes in FECD revealed the PI3K-AKT, TNF, AGE-RAGE, and focal cell adhesion formation pathways as having the greatest number of potential target genes (10, seven, seven, and seven genes, respectively) in the FECD group (Figure 3 and Figure 4). These pathways were prioritized because they showed the largest numbers of candidate target genes among the retained miRNA–target pairs and are each linked to processes previously implicated in FECD pathophysiology, including oxidative stress, inflammatory signaling, and endothelial cell adhesion.

### 2.4. Comparison with Published Data

**Figure 5:**
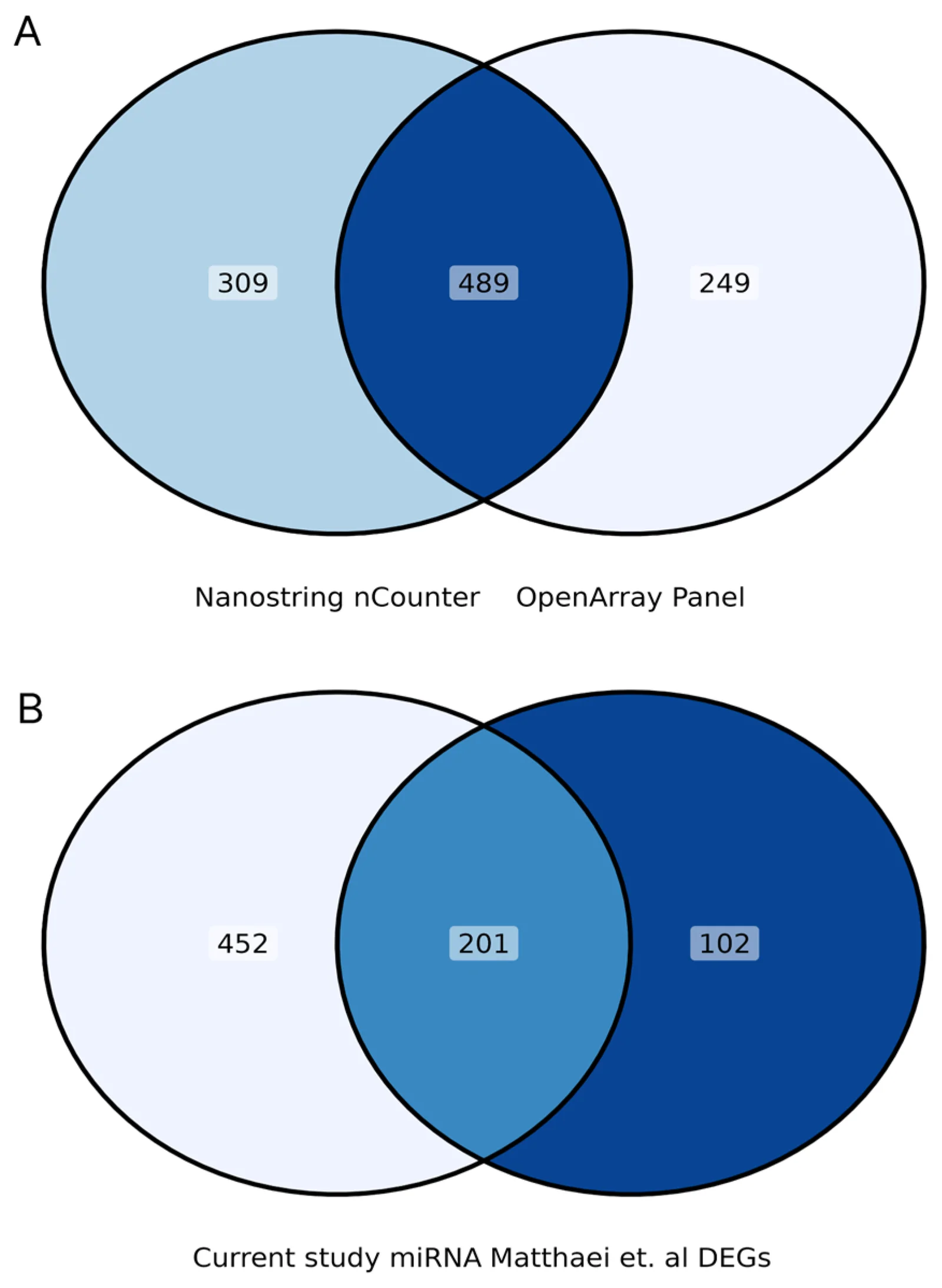
Array comparison between current study and [45]. (**A**) Common and unique microRNA sets between the Nanostring nCounter Human v3 miRNA panel (current study) and the TaqMan OpenArray Human MicroRNA Panel [45]. (**B**) Common and uniquely expressed detectable microRNAs in the compared studies.

To assess the reproducibility of miRNA expression changes, we compared our results with the differentially expressed miRNA data from [45]. Comparison resulted in at least 201 expressed miRNA among assays and 554 total unique for both assays (Figure 5B).

**Figure 6:**
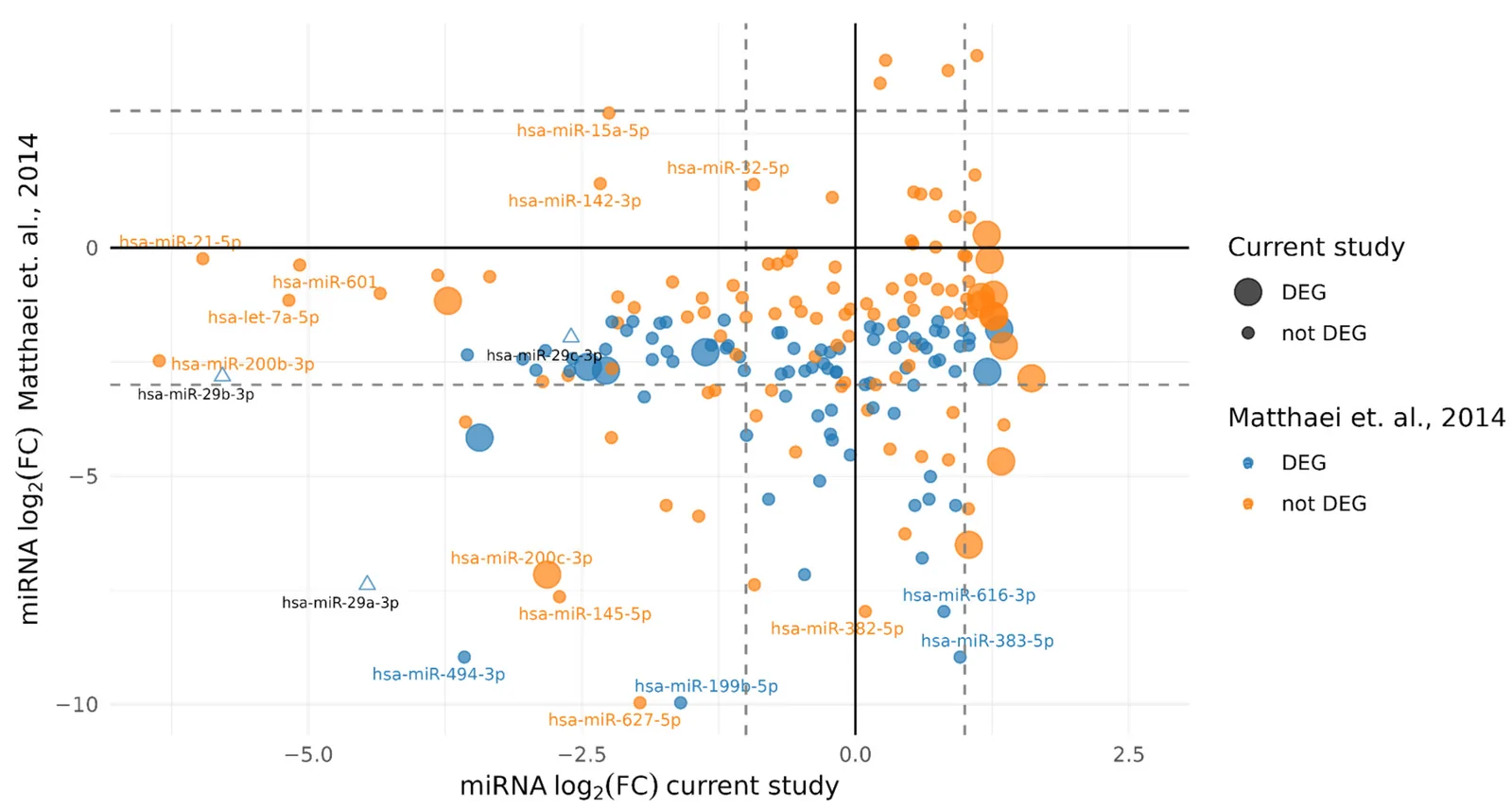
Similarity and dissimilarity of differentially expressed miRNAs in our dataset and the comparison dataset from [45]. Only 6 miRNAs were significantly differentially expressed in both studies. Dotted line set on |log2FC| = 1.

A comparison of the differential expression levels for the common miRNAs among studies revealed that the majority of these miRNAs are near the detection threshold of the panels and are panel-specific. They exhibit a constant Fold Change level in one panel, compared to a variable level in the other, so the pattern is alongside the axis (Figure 6). A subset of miRNAs demonstrates an opposite direction of expression, indicating incomplete reproducibility of the result and discordance in miRNA expression.

Of the 201 miRNAs detected in both datasets, six were reported as significantly differentially expressed in both studies (Figure 6); six are presented in Table 3. In the correlation analysis, hsa-miR-30d-5p was negatively correlated with *PPP1R15B* and *SLC7A5*, and hsa-miR-576-3p with *GBP4*. These associations identify candidate interactions rather than demonstrating regulation in FECD.

**Figure 7:**
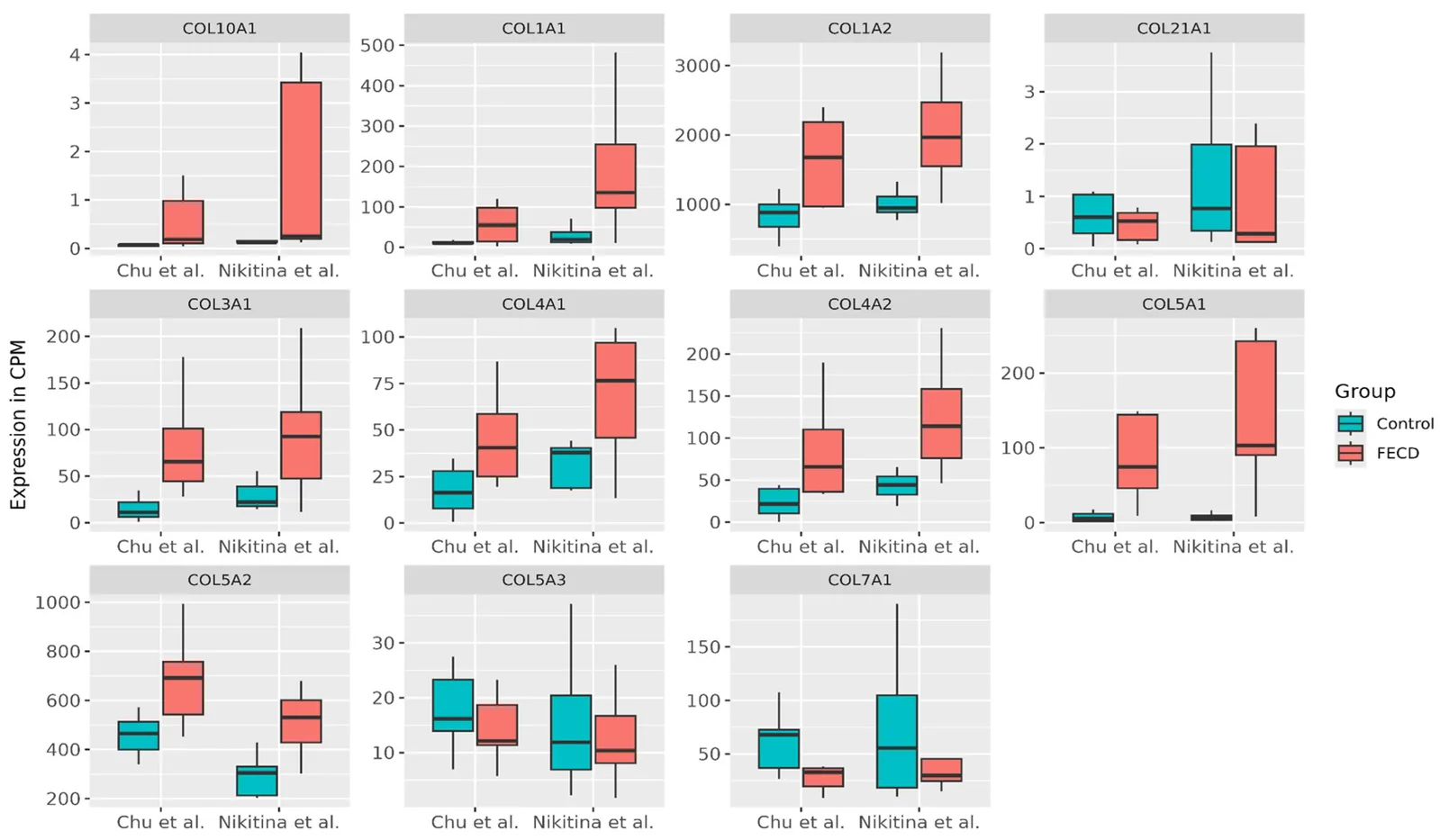
Expression levels of probable hsa-miR-29 target collagen genes from [45] according to the total RNA-seq data from [24,50].

Some of the microRNAs previously discussed in the literature, like hsa-miR-29a/b/c, are also represented in our data with high Fold Change but low *p*-value-adjusted (FDR) significance. According to our data, hsa-miR-29 is also decreased in FECD, and its possible targets, COL1A1 and COL4A1, show correspondingly higher expression levels, which is consistent with the latest reports (Figure 7). However, because the hsa-miR-29 family did not meet the prespecified FDR significance threshold and was not retained among the correlated miRNA–target pairs, these observations should be regarded as suggestive and hypothesis-generating rather than as confirmed regulatory relationships.

**Figure 8:**
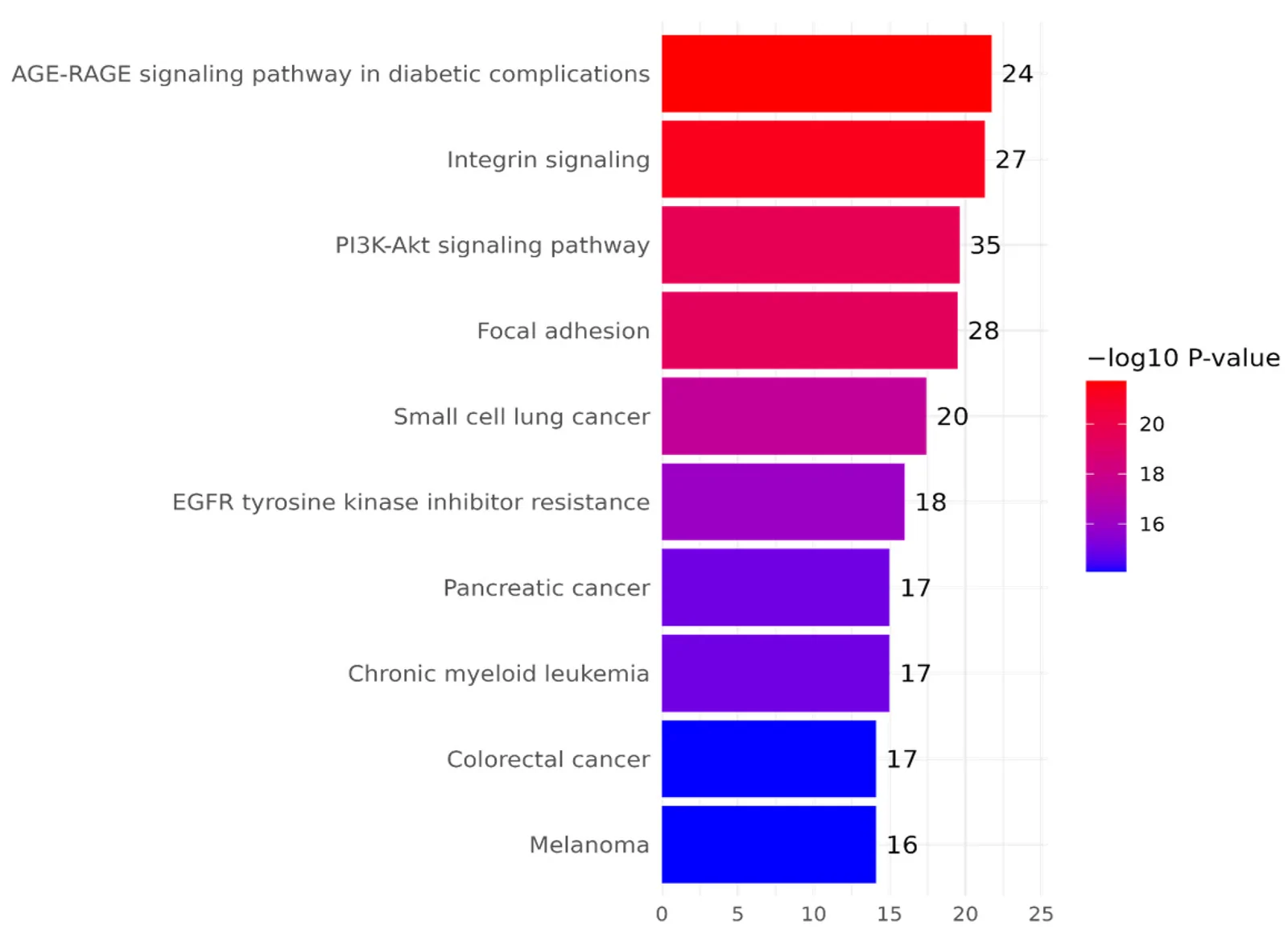
KEGG enrichment of hsa-miR-29 family target genes. The digit on columns indicates the number of genes found in pathways. To address reproducibility issues, we compare DICER-related genes’ expression in two analyzed datasets.

KEGG enrichment of hsa-miR-29 family target genes share a lot of pathways with negatively correlated miRNA–target gene pairs from the current study, especially in AGE-RAGE signaling pathway, PI3K-Akt signaling, and focal adhesion pathways (Figure 8). The overlap in pathway annotations provides a functional context for further investigation but does not establish coordinated or synergistic regulation by miR-29 and the miRNAs identified in our correlation analysis.

**Figure 9:**
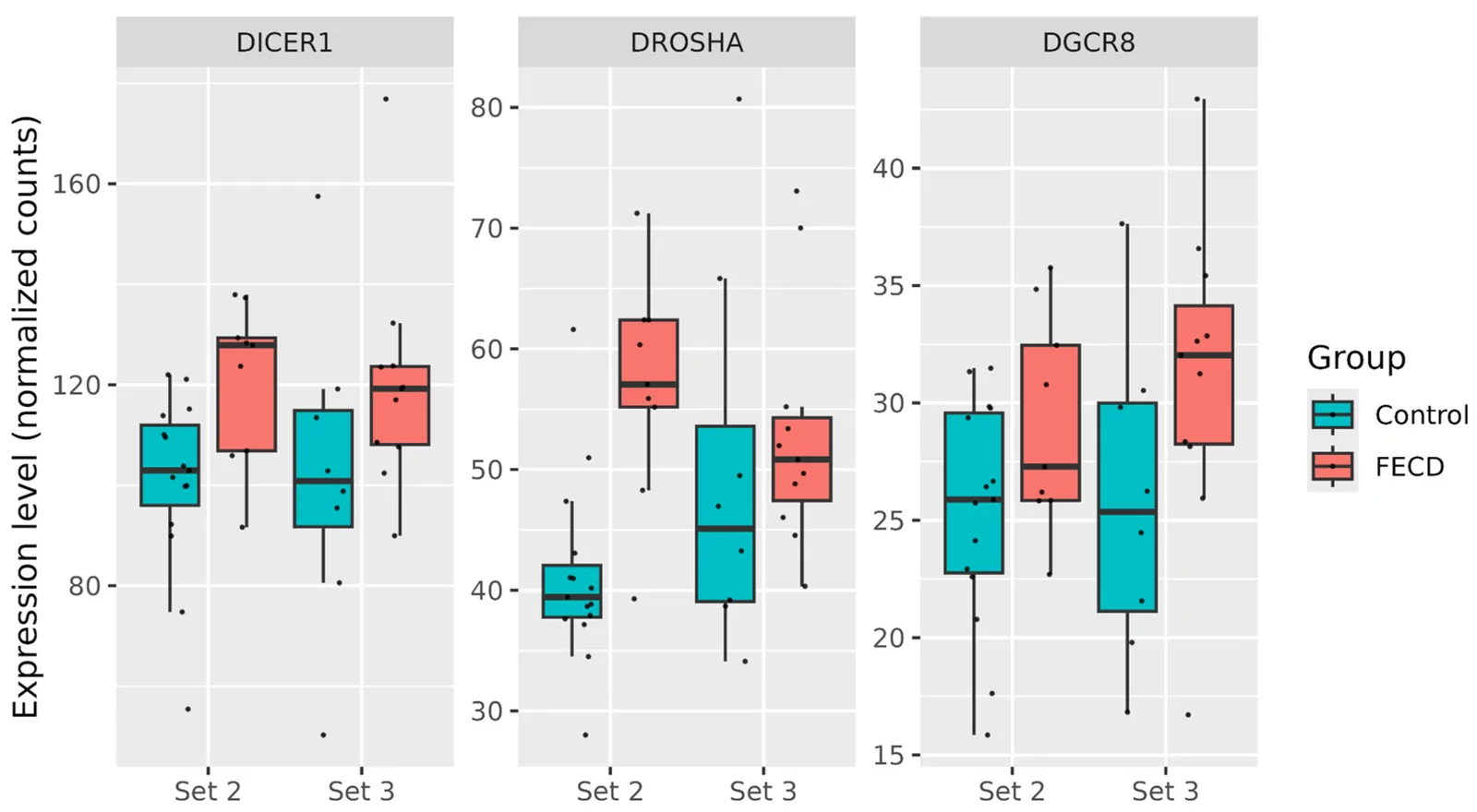
RNA CPM expression level of genes involved in miRNA biogenesis. CPM expression in *DICER1*, *DROSHA*, and *DGCR8*. Sets 2 and 3 are from [24,50], respectively.

At the transcript level, *DICER1*, *DROSHA*, and *DGCR8* mRNAs are not downregulated, in contrast to the downregulation reported by [45] (Figure 9). These transcript-level observations do not, by themselves, establish changes in protein abundance or miRNA-processing activity.

## 3. Discussion

MicroRNAs are transcribed from the genome as primary miRNAs (pri-miRNAs), which are processed in the nucleus by the Drosha complex into precursor miRNAs (pre-miRNAs). These pre-miRNAs are then exported to the cytoplasm and further cleaved by the enzyme Dicer into mature miRNA duplexes. One strand of this duplex is incorporated into the RNA-induced silencing complex (RISC). Within RISC, miRNAs guide the complex to partially complementary sequences, primarily in the 3′ untranslated regions of target mRNAs. This binding typically results in either translational repression or degradation of the target mRNA, thereby fine-tuning protein production. Since their discovery, thousands of miRNAs have been identified and researched for their roles in normal biology and disease. This specificity and sensitivity of miRNAs make them attractive non-invasive biomarkers for detecting various disorders.

In this work, we have conducted miRNA profiling of corneal endothelium samples of FECD patients and controls. Differentially expressed miRNAs were estimated. Among the downregulated miRNAs in FECD were miR-630, miR-1972, and miR-1285. There are no direct references in the literature about the role in FECD pathogenesis for these miRNAs. However, there is information about their role in different disease pathogeneses. It is known that miR-630 is induced by oxidative stress in lens epithelial cells, and it suppresses their migration by targeting *ALCAM* [51]. In cataract, the upregulation of miR-630 leads to the suppression of proliferation and induction of apoptosis in lens epithelial cells via targeting *E2F3* [52]. It is known that oxidative stress is upregulated in FECD, and if miR-630 is induced by oxidative stress, it should be upregulated. But it is not, and thus miR-630 possibly does not have a connection with oxidative stress in corneal endothelium, especially considering the preserved expression of the *ALCAM* gene. In light of these findings, miR-630 in FECD may attenuate apoptotic regulation and promote EMT/fibrotic changes. It is known that migration of cells is activated in FECD. Thus, the downregulation of miR-630 may be involved in the regulation of migration activity.

Also among miRNAs downregulated in FECD, we found miR-1972. It has been reported to be significantly (21-fold increase) upregulated in the corneal epithelium of keratoconus patients, suggesting a potential role in corneal pathophysiology [53].

There is information about the role of miR-1285 in a conjunctival model of scarring, which was found to be downregulated in FECD. Earlier, it was reported that miR-1285 can suppress *p53* and is involved in the regulation of EMT markers by targeting *TGM2* during inflammation [54].

Among significantly upregulated miRNAs in FECD corneal endothelium samples, there were miR-665-3p and miR-302b. MiR-665-3p was studied in a mouse model of fungal keratitis. It was upregulated during Fusarium solani infection, and its inhibition enhanced autophagy and reduced corneal inflammation [55]. It appears to enhance the inflammatory response, so it may indicate enhanced inflammatory signaling. This is in alignment with findings that inflammation is upregulated in FECD.

The miR-302 family are known regulators of pluripotency and cell renewal that target genes involved in AKT signaling, senescence, and stress response [56]. Our data revealed that miR-302b was significantly upregulated. Given its high homology with the well-studied miR-302a, which is known to enhance corneal endothelial regeneration by reducing senescence and endoplasmic reticulum stress, this suggests a shared therapeutic potential for protecting corneal cells against pathological damage [57].

According to mirTarBase, the hsa-miR-29 family targets a lot of ECM components’ proteins that are dysregulated in FECD tissue. Expression level of various collagens and laminins (*COL1A1*, *COL3A1*, *COL4A1*, *COL4A2*, *COL5A1*, *COL10A1*, *LAMC1* and *LAMC2*) may lead to excessive accumulation and thickening of the basement membrane and formation of a pathologically dense basal layer, as shown by Kenney et al. in 1984 [58]. Upregulation of the LOX family may increase the rigidity of the matrix with fibrotic changes driven by SPARC, FBN1, and a disruption of cell adhesion by ADAM12. Transfection with a miR-29b resulted in reduced expression of a majority of collagen genes and *ADAM12*, giving more evidence to the idea that the miR-29 family is able to modulate ECM changes [59]. Potential targets are involved in the regulation of COL18A1, COL4A2, SLC1A2, SLC18A2, SLC40A1, SLC7A2, and SLC7A5, as well as various components of the endothelium, and genes that modulate Inflammation and immune response (*CLEC4A*, *LY75*/*CD302*, *IL1RAP*, *SOCS3*, and *TLR7*), and they are also involved in the regulation of cell growth and apoptosis (CCND1, CDKN2B, BCL2L1, and TNFRSF11B).

The PI3K-Akt pathway regulates cell survival, proliferation, and metabolism; its dysfunction may contribute to corneal endothelial apoptosis and barrier impairment. Dysregulation of this pathway, often triggered by oxidative stress and reactive oxygen species (ROS), has been implicated in pathological endothelial cell loss and compromised barrier-function characteristics in FECD [60].

The focal adhesion pathway is critical for corneal endothelial cell adhesion, migration, and signal transduction. In the corneal endothelium, focal adhesions regulate cellular attachment to Descemet’s membrane and coordinate cytoskeletal dynamics required for wound healing and response to mechanical stress [61].

TNF and AGE–RAGE signaling have previously been implicated in FECD by transcriptomic pathway analyses, alongside alterations in oxidative stress- and immune response-related processes [60].

One of the first studies addressing miRNA regulation problems in FECD was published in 2014 by Matthaei et al., with TaqMan OpenArray Human MicroRNA Panel. Comparison with this resulted in 489 miRNAs present in both arrays, and 309 and 249 miRNAs presented uniquely in Nanostring and OpenArray, respectively. Differences in microchips allow for comparison and expansion of research results. It is noteworthy that significant differences in DE miRNA fold changes were observed between the two studies. In our data, 79 miRNAs were upregulated, and 19 were downregulated, whereas in the study by [45], all reported miRNAs showed downregulation. This discrepancy may be attributed to differences in cohort composition and tissue procurement, profiling platforms and normalization procedures, sample processing, analytical approaches, or other hidden factors, highlighting the importance of methodological consistency in miRNA research. Our analysis does not reproduce the DICER1-based miRNA downregulation in FECD endothelium reported by [45]: the change in DICER1 expression in our cohort is not significant, and we found no evidence that the overall miRNA level in FECD is downregulated. However, given the differences in cohorts and platforms between the studies, this should not be taken as evidence against the original findings. Overall, at the transcript level, our analysis revealed higher DICER1 mRNA abundance, in contrast to the downregulation reported by [45] (Figure 9); this observation is restricted to transcript abundance and does not establish altered DICER1 protein levels or miRNA-processing activity. This discrepancy may indicate the involvement of more complex regulatory mechanisms, potentially including other unknown factors. The microRNAs hsa-miR-29a, -b, and -c, which have been previously reported in the literature, also showed high Fold Changes in our dataset, although the corresponding adjusted *p*-values (FDR) did not reach statistical significance. However, collagen genes are already substantially overexpressed in FECD, as supported by RNA-seq data from Chu et al. in 2020 and Nikitina et al. in 2019 (Figure 7), and the contribution of miR-29 to this increase could not be confirmed in our dataset [24,50]. Moreover, the absence of the hsa-miR-29 family from the correlated pairs indicates that its involvement in FECD remains suggestive rather than confirmed.

Several limitations of this study should be acknowledged. The FECD and control groups differed substantially in regard to sex distribution, age, and tissue procurement (surgical specimens versus postmortem donor tissue), and disease status was partially confounded with the NanoString assay run; these factors cannot be fully disentangled in a cohort of this size. The identified miRNA expression changes should therefore be interpreted as differences between the observed groups rather than as definitively FECD-specific effects. Given the pronounced sex imbalance and the limited sample size, age and sex were not included as covariates in the primary differential-expression model; instead, sensitivity analyses with limma models adjusted for age and sex are reported in the Supplementary Materials and acknowledged here as major limitations. In addition, the correlation analysis was based on eight specimens per group; the reported miRNA–target pairs should therefore be regarded as exploratory candidate associations rather than evidence of direct regulation, and their stability to individual observations could not be formally assessed. Finally, transcript-level changes in miRNA biogenesis genes do not establish changes in protein levels or processing activity. Overall, the present work should be viewed as an exploratory, hypothesis-generating study that requires replication in larger and better-balanced cohorts.

## 4. Materials and Methods

### 4.1. Tissue-Specimen Collection

This study included patients diagnosed with FECD and donor samples. The diagnosis of FECD was established on the basis of anamnesis collection results and a thorough ophthalmological examination of the anterior segment of the eye. The primary diagnostic criterion was the presence of corneal guttae (cornea guttata). All patients underwent slit-lamp biomicroscopy using an SL-30 (Opton, Oberkochen, Germany) and confocal microscopy using an SP-2000P (Topcon, Tokyo, Japan). Patients referred for keratoplasty surgery and who permitted the preservation of corneal endothelium samples excised during the surgery were included in this work. All patients underwent Descemet Membrane Endothelial Keratoplasty (DMEK). The dissected Descemet’s membranes with corneal endothelium were placed into a microtube containing the preservation solution RNAlater (ThermoFisher Scientific; Waltham, MA, USA). The samples were then preserved according to the manufacturer’s instructions. Venous blood was collected from patients with FECD for DNA extraction.

Control samples of corneal endothelium were obtained from the eye bank of the S.N. Fyodorov Eye Microsurgery Federal State Institution. Donor samples unsuitable for corneal transplantation were selected for this study. Collection was performed under sterile operating-room conditions. The excised corneal endothelium sample on Descemet’s membrane was preserved in RNAlater, similarly to how it was performed for patient samples. For donor samples, iris fragments were collected as a source of DNA.

More detailed information on the sample-collection procedure can be found in our previous publication concerning these corneal endothelial samples [50].

### 4.2. Genotyping of CTG18.1 Repeats

To determine patients’ expansion status, genotyping all participants for the CTG18.1 repeat expansion marker is essential. Based on genotyping results, patients were divided into groups with and without the trinucleotide repeat expansion. Donor-tissue genotyping confirmed the absence of trinucleotide repeat expansions. An expansion was defined as >40 CTG18.1 repeats. Short tandem repeat analysis and triplet-primed PCR followed by fragment analysis were used to determine repeat numbers. Reaction conditions, compositions, and result interpretation followed protocols done as described [62].

### 4.3. miRNA Isolation

The fraction of short RNAs was isolated simultaneously with the isolation of total RNA. Total RNA was isolated using the RNeasy Micro Plus Kit (Qiagen, Hilden, Germany). Descemet’s membranes were lysed in the RLT Plus buffer, with beta-mercaptoethanol added. The first stage involved disruption and homogenization of the sample using the TissueRuptor II manual homogenizer (Qiagen, Hilden, Germany) and disposable probes for it. The homogenization time was 20–30 s. Precipitation of debris was performed by centrifugation for 2 min at 13,500 rpm (Minispin, Eppendorf, Hamburg, Germany). The supernatant was applied to the gDNA Eliminator Spin column and centrifuged for 15 s at 13,500 rpm. The eluate was mixed with 70% ethanol, according to the manufacturer’s instructions, and applied to a RNeasy Mini Spin column. The column was centrifuged for 15 s at 13,500 rpm. Long RNAs (>200 nucleotides) were bound with the column during this procedure. The eluate containing RNA fractions shorter than 200 nucleotides was collected. An additional amount of 96% ethanol (×0.65 V) was added to the eluate. This mixture was loaded onto a new RNeasy MinElute column from the RNeasy MinElute Cleanup Kit (Qiagen, Hilden, Germany) and centrifuged for 15 s at 13,500 rpm; afterward, the eluate was discarded. Washing was performed with 500 uL of RPE buffer from the kit, and after being centrifuged for 15 s at 13,500 rpm, the eluate was discarded. The final wash was performed with 80% ethanol. The column was centrifuged for 15 s at 13,500 rpm, and the eluate was discarded. Drying of the membrane on the column was performed by centrifugation of the empty column for 1 min at 13,500 rpm. The fraction of short RNAs was eluted from the column in 20 mcl of RNAse-free water. These procedures were conducted in accordance with protocol RY26, available on the Qiagen website.

### 4.4. NanoString nCounter miRNA Profiling

For miRNA expression analysis, the following 27 corneal endothelium samples were initially selected: 16 with Fuchs’ dystrophy (including 11 with CTG18.1 repeat expansion in *TCF4* and 5 without it) and 11 control samples. The final miRNA analysis dataset therefore comprised 14 FECD and 8 control samples (Supplementary File S2; Supplementary File S1). The collected sample set showed gender imbalance because control donors were obtained postmortem (mostly from accident victims) and were predominantly male. Samples containing microRNA were processed using nCounter cell-free miRNA profiling with nCounter Human v3 miRNA Expression Assay (NanoString, Seatlle, WA, USA).

### 4.5. RNA-Seq Data Preparation

For analysis, a paired set of transcriptomics data was taken, obtained from the same RNA aliquots from which microRNA was isolated (PRJNA524323 [50]). Quality control on raw reads using FastQC (v0.12.1) [63] and MultiQC (v1.15) [64] was done both before and after adapter trimming with cutadapt (v3.3) [65] and quality filtering with Trimmomatic (v0.39) [66]. Pseudoalignment was executed using Salmon (v1.10.2) [67] against the GRCh38 reference and Gencode v43 transcriptome. Gene-level summarization was performed with tximport (v1.26.1) [68] by employing length-scaled TPM metrics while disregarding transcript versions. Expression levels for genes were generated by edgeR (v3.28.1) [69], using TMM scaling factors and filtering for genes with counts > 2 at least in one sample. CPM values from edgeR were utilized in subsequent analyses. Data were visualized using ggplot2 (v3.5.2) [70].

### 4.6. miRNA Data Analysis

The obtained miRNA expression data were preprocessed in nSolver software v4.0.7 (NanoString) using the ligation-normalization method according to the manufacturer’s protocol, without background correction. All samples successfully passed nSolver’s quality control. The principal component analysis with PCAtools (v2.10.0) [71] on log2-transformed data after removing genes with the lowest 10% variance was used to assess data heterogeneity. To filter non-zero expressed miRNA from the data, the empirical cutoff was set on mean expression of 2.2 in either the FECD orcontrol groups.

Prior to further analysis, all miRNA names were manually converted to HGNC symbol nomenclature to obtain genomic location information (for subsequent genomic context analysis). Strand-specific annotation (5P/3P) was obtained by cross-referencing the miRNA expression table with both Genecode v43 annotation and MIMAT ID-specific annotation from the Mirbase website. Deprecated in Genecode v43, miRNAs were removed from analysis. For miRNAs with compound names of type MIR + MIR (e.g., hsa-miR-106a-5p + hsa-miR-17-5p), only the first miRNA matching the manufacturer-provided MIMAT ID was retained for downstream analysis. Genome coordinates of miRNA from assay were obtained this way and strand-specific intersecting was performed via bedtools (v2.30.0) [72] to obtain miRNA’s host genes, where they co-express as mirtrons.

### 4.7. Differential Expression

Differential expression of microRNAs between the control and FECD groups was calculated using three methods to achieve maximum testing rigor: Student’s *t*-test, the Mann–Whitney test, and linear regression with the limma package (v3.58.1) [73]. The resulting microRNAs were filtered for significance (*p*-adjusted FDR < 0.05) and for |log2FC| > 1). This resulted in 98 identified differentially expressed microRNAs being presented in the Supplementary Materials. To assess the potential effects of age and sex on the differential-expression results, sensitivity analyses were additionally performed using four limma models: (1) an unadjusted model including FECD status only, (2) a model adjusted for sex, (3) a model adjusted for age, and (4) a model adjusted for both sex and age. The numbers of differentially expressed miRNAs identified by these models were 178, 48, 169, and 14, respectively; the full lists are provided in the Supplementary Materials. Adjustment for age alone resulted in a number of discoveries similar to the unadjusted analysis, whereas adjustment for sex substantially reduced the number of differentially expressed miRNAs, which should be interpreted in the context of the pronounced sex imbalance between the groups.

### 4.8. Correlations

For correlation analysis, transcriptomic data from samples with available datasets were utilized, comprising 8 FECD and 8 control samples. Analyses were performed separately for control and FECD groups. To reduce noise in gene expression, near-zero values resulting from log-transformation were removed.

Spearman rank correlation between gene and miRNA expression matrices was calculated. Based on the correlation values’ distribution, a cutoff of |0.7| was applied. Pairs with correlations > |0.7| were selected, prioritizing negative correlations due to their potential for miRNA-mediated gene suppression. Correlated pairs were filtered against experimental-only-validated miRNA–target gene interactions found in miRTarBase v10 [74].

Permutation-based *p*-values were computed, due to limited sample size, with false discovery rate adjustment. To prioritize biologically consistent signals over dataset-specific outliers, we focused on RNA transcripts exhibiting concordantly high expression in control samples from both our primary dataset, [50], and the [24] dataset. This tri-dataset validation strategy required elevated expression in our controls; [24] controls ensured selected miRNA–target genes pairs represent robustly expressed targets suitable for potential experimental validation. Final pair filtration used log Fold Change (FECD vs. control) and required [24] CPM expression > 2 with |log2FC| > 1.

### 4.9. KEGG Enrichment

For functional interpretation of the identified genes, an analysis was performed using the KEGG (Kyoto Encyclopedia of Genes and Genomes) [75] database. The obtained genes were compared with KEGG maps using the clusterProfiler (version 3.0.4) [76] and visualized by pathview v1.42.0 [77] packages to determine their involvement in signaling pathways.

## 5. Conclusions

FECD is a complex disease, uniting various factors from ECM thickening, EMT-derived changes, and increased transcription noise. Therefore, it remains unclear how central the role of microRNAs is in FECD pathology, whether they act as important cofactors of the disease or are mostly a consequence of broader pathological changes. The literature is fragmented: some studies on individual miRNAs are consistent, while others contradict each other. Even when compared to the recent work of [45], clear biases and poor reproducibility can be seen, partly due to differences in assay platforms, compared to RNA-seq data. Still, several earlier findings have been confirmed, and the candidate miRNA–target relationships identified here may inform future functional studies and, ultimately, therapeutic exploration. The cornea endothelium is a tiny, cell-poor, challenging tissue to work with, making it difficult to confidently assess RNA and miRNA extraction quality without the after-use of RNA-seq data. A full transcriptome profile is essential to control for potential laboratory artifacts, such as accidental contamination from blood vessels or other cell types, which could otherwise compromise the data integrity. This inherent difficulty is compounded by the inherent variability of miRNA expression profiles and their challenging detection. Together, these factors make such studies particularly demanding and underscore the necessity for replication to ensure the generation of robust and biologically meaningful results. Given the limitations of the current cohort, the findings of this study should be regarded as exploratory and hypothesis-generating, requiring replication in larger and better-balanced cohorts.

## Figures and Tables

**Table 1:** Group metadata. Data are presented as mean ± standard deviation.

		Males	Females	Total
FECD	Samples	2	12	14
	Age, years, mean (SD)	69 (7.1)	67.2 (11)	67.4 (10.3)
	Samples with CTG18.1 > 40	1	10	11
Control	Samples	6	2	8
	Age, years, mean (SD)	59 (4.7)	55.5 (12.0)	58.1 (6.2)
	Samples with CTG18.1 > 40	0	0	0

**Table 2:** Top nine negatively correlated miRNA–target gene pairs in the FECD group.

Target Gene Symbol	miRNA	MIMAT ID	Correlation
MMP16	hsa-miR-1197	MIMAT0005955	−0.976
FMN1	hsa-miR-520e	MIMAT0002825	−0.905
MAP3K8	hsa-miR-375	MIMAT0000728	−0.905
TMOD1	hsa-miR-544a	MIMAT0003164	−0.905
FMN1	hsa-miR-367-3p	MIMAT0000719	−0.886
FMN1	hsa-miR-520d-3p	MIMAT0002856	−0.886
TNFAIP3	hsa-miR-626	MIMAT0003295	−0.886
ADM	hsa-miR-26a-5p	MIMAT0000082	−0.881
ATP11A	hsa-miR-769-5p	MIMAT0003886	−0.881

**Table 3:** miRNA differentially expressed in both [45] and current study.

miRNA	log2FC	*p*_adj	Normalized Counts, Mean	
			FECD Group	Control Group
hsa-miR-103a-3p	1.31	9.96 × 10^−4^	2.49	1.00
hsa-miR-151a-3p	−1.37	2.98 × 10^−2^	3.31	8.57
hsa-miR-181a-5p	−3.43	2.98 × 10^−2^	3.11	33.60
hsa-miR-186-5p	−2.28	4.35 × 10^−2^	3.95	19.19
hsa-miR-30d-5p	−2.44	4.35 × 10^−2^	4.31	23.47
hsa-miR-576-3p	1.21	2.96 × 10^−2^	2.67	1.16

## Data Availability

RNA sequencing data from ref. [50] are available at NCBI Sequence Read Archive under Accession No. PRJNA524323. RNA sequencing data from ref. [24] are available at Gene Expression Omnibus (GEO), accession number GSE142538. Differentially expressed miRNA from ref. [45] (https://doi.org/10.1167/iovs.13-12689) was taken from article Supplementary File S2. Raw counts from the current study are presented in Supplementary File S6.

## Supplementary Materials

The following supporting information can be downloaded at https://www.mdpi.com/article/10.3390/ijms272110044/s1.
