# Supplementary material for: Integrated microRNA and mRNA Profiling in the Corneal Endothelium of Fuchs Endothelial Corneal Dystrophy
Source: Int J Mol Sci. 2026 Nov 9;27(21):10044. doi: 10.3390/ijms272110044 (PMC13764593; doi:10.3390/ijms272110044)
Supplement: Supplementary file 1 [file ijms-27-10044-s001.zip › ijms-4581850-supplementary/Supplementary File 5_plots.docx]

### 1. PCA with additional cofactors
